# A Prize

**Published:** 1847-06

**Authors:** 


### ARTICLE VIII.

### A PRIZE.

We observe that the Medical Society of Lexington, Kentucky, offers a prize of fifty dollars, or a gold medal worth that sum, at the option of the receiver, for the best inaugural thesis that shall be presented to the Faculty of Transylvania University, for the Degree of Doctor of Medicine, at its next session.

